# Lifestyle and clinical risk factors in relation with the prevalence of diabetes in the Indonesian urban and rural populations: The 2018 Indonesian Basic Health Survey

**DOI:** 10.1016/j.pmedr.2024.102629

**Published:** 2024-01-24

**Authors:** Farid Kurniawan, Fathimah S. Sigit, Stella Trompet, Em Yunir, Tri Juli E. Tarigan, Dante S. Harbuwono, Pradana Soewondo, Dicky L. Tahapary, Renée de Mutsert

**Affiliations:** a Division of Endocrinology, Metabolism, and Diabetes, Department of Internal Medicine, Dr. Cipto Mangunkusumo National General Hospital/Faculty of Medicine Universitas Indonesia, Jakarta, Indonesia; b Department of Parasitology, Leiden University Medical Center, Leiden, The Netherlands; c Metabolic Disorder, Cardiovascular, and Aging Research Cluster, The Indonesian Medical Educational and Research Institute, Faculty of Medicine Universitas Indonesia, Jakarta, Indonesia; d Department of Public Health Nutrition, Faculty of Public Health Universitas Indonesia, Jakarta, Indonesia; e Department of Internal Medicine, Section of Gerontology and Geriatrics, Leiden University Medical Center, Leiden, The Netherlands; f Department of Clinical Epidemiology, Leiden University Medical Center, Leiden, The Netherlands

**Keywords:** Urban/rural, Urbanization, Indonesia, Diabetes, Lifestyle factors, Clinical factors

## Abstract

**Aims:**

To investigate the differences between Indonesian urban and rural populations in the association of lifestyle and clinical factors with diabetes prevalence.

**Methods:**

Using database of the 2018 Indonesian Basic Health Survey, which was conducted in April-May 2018, non-pregnant respondents aged ≥15 years old with available blood glucose data (n urban = 17,129, n rural = 16,585) were included in this study. The diagnosis of diabetes was based on the combination of known diabetes, i.e., a previous history of diabetes or use of anti-diabetes medication, and unknown diabetes based on blood glucose criteria. We performed logistic regression analyses separately for the urban and rural populations to examine the association of lifestyle and clinical factors with prevalent diabetes.

**Results:**

Indonesian urban population was less physically active, had a lower proportion of adequate fruit and vegetable intake, and had higher individuals with obesity than rural population. Although there were no differences in the total prevalence of diabetes between the two populations (10.9 % vs. 11.0 %, for urban and rural, respectively), the prevalence of known diabetes was twice higher in urban than in rural population (3.8 % vs. 1.9 %). Physical activity was associated with lower risk of diabetes, especially in the urban population [prevalence OR (95 %CI): 0.91 (0.85; 0.98) for urban and 0.94 (0.89; 1.00) for rural). Obesity, hypertension, and dyslipidemia were risk factors for prevalent diabetes in both populations.

**Conclusions:**

Indonesian rural population showed relatively better lifestyle and clinical profiles compared to their urban counterparts. However, no differences were observed between the two populations in the relation between risk factors and diabetes. Special attention needs to be addressed to the high prevalence of undiagnosed and untreated diabetes in Indonesia.

## 1 Introduction

The prevalence of diabetes is increasing worldwide, from 8.3 % in 2011 to 10.5 % in 2021, and is projected to become 12.2 % in 2045 (IDF Diabetes Atlas 10th edition. International Diabetes Federation, 2021). Currently, more than 80 % of people with diabetes live in low and middle-income countries (LMICs), and the greatest relative increase in diabetes prevalence is expected to occur in middle-income countries (IDF Diabetes Atlas 10th edition. International Diabetes Federation, 2021, Sun et al., 2022). Indonesia, as one of the LMICs with more than 19 million people suffering from diabetes in 2021, ranked as the 5th highest country of people with diabetes in the world (IDF Diabetes Atlas 10th edition. International Diabetes Federation, 2021).

Diabetes causes significant morbidity and mortality and is an established risk factor for other diseases, such as cardiovascular diseases, end-stage renal diseases, and cancers (Lin et al., 2020). In 2016, diabetes became the third leading cause of disability-adjusted life year in Indonesia (Mboi et al., 2018), and has become a national economic burden due to its high healthcare costs (Hidayat et al., 2022).

The worldwide prevalence of diabetes was estimated to be higher in urban (12.1 %) than in rural (8.3 %) areas (Sun et al., 2022). The advancing socio-economic development in many LMICs that promote rapid urbanization and influence the environmental and social changes, may contribute to this trend (Fan et al., 2019). Previous study has linked urbanization with unhealthy dietary habits and reduced physical activity, resulting in a surplus of energy stored as body fat (Hall et al., 2012). This may result in obesity and consequent low-grade inflammatory state and insulin resistance, pathways leading to type 2 diabetes (T2D) (Schuster, 2010). Our previous study in Indonesian young adults showed a higher prevalence of obesity in the urban compared to rural population (Kurniawan et al., 2022).

Besides obesity, previous studies also showed that hypertension and dyslipidemia differed greatly in prevalence between rural and urban populations (Wang et al., 2018, de Groot et al., 2019). Apart from these lifestyle and biological factors, differences in the level of education, type of employment, and socio-economic status are often observed between urban and rural populations and could potentially influence the incidence of diabetes (Kyrou et al., 2020). Based on the 2014 Indonesian Family Life Survey, the prevalence of diabetes in Indonesia is 7,2% with lower educational levels, unemployment, higher age and body mass index, hypertension, and urban childhood residence were associated with diabetes (Indrahadi et al., 2021, Mulyanto et al., 2019, Tanoey and Becher, 2021).

We hypothesized that these urban–rural discrepancies in lifestyle, clinical, and socio-demographic factors contribute to the differences in the prevalence of diabetes between these two populations. Therefore, the aim of our study was to investigate the differences in these risk factors between Indonesian urban and rural populations and their relationship with the prevalence of diabetes (Suppl Fig. 1).

## 2 Methods

### 2.1 Study design and population

This cross-sectional study utilized the 2018 Indonesian Basic Health Survey (Riset Kesehatan Dasar, RISKESDAS) data, a comprehensive survey assessing communicable and non-communicable diseases in Indonesia, and their associated risk factors. This survey was commenced in April-May 2018 according to the sampling frame determined by the Indonesian Central Bureau of Statistics (Biro Pusat Statistik/BPS) that was used in the National Socio-economic Survey (Survei Sosial Ekonomi Nasional/SUSENAS) on March 2018.

**Fig. 1:**
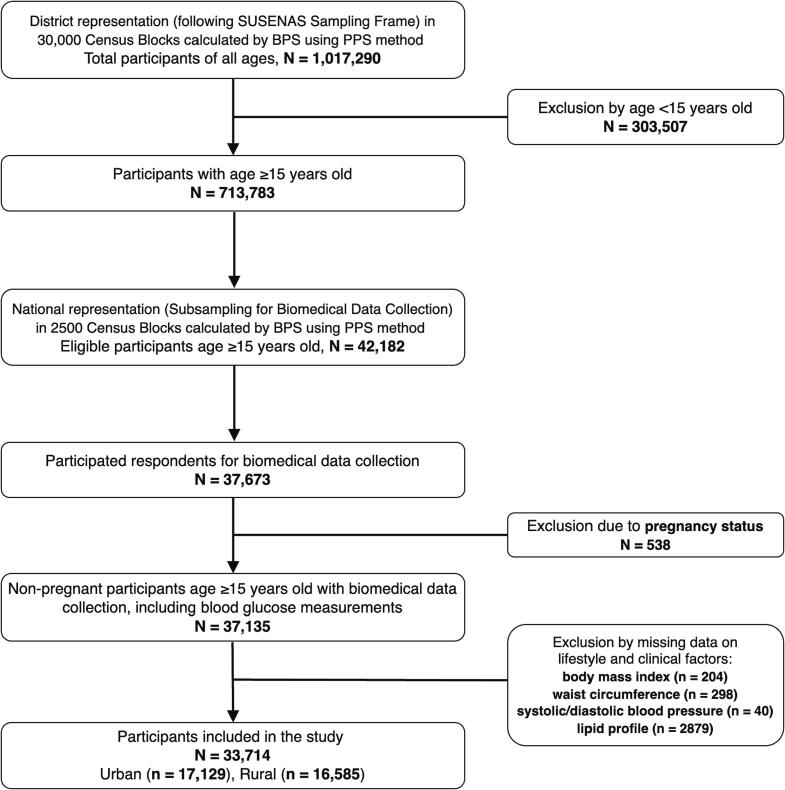
Flow chart for the inclusion of study participants obtained from the 2018 Indonesian Basic Health Survey database. SUSENAS: Survey Sosio-ekonomi Nasional/National Socio-economic Survey; BPS: Biro Pusat Statistik/Indonesian Central Bureau of Statistic.

A detailed explanation of the methodological sampling of the 2018 RISKESDAS has been described previously (Dany et al., 2020). Briefly, a stratified, multistage systematic random sampling design and probability proportional to size (PPS) method with consideration of urban–rural distribution resulting in 30,000 census blocks, were used to select households and participants from 34 provinces in Indonesia. This survey population was 1,017,290 individuals of all ages, with 713,783 aged ≥15 years. Up to 2500 census blocks across 26 provinces were sub-sampled in representing national level for biomedical data collection, including blood glucose measurement. From the 42,182 eligible respondents aged ≥15 years calculated using the PPS sampling method, 37,673 participated in the biomedical data collection. Pregnant women and individuals with missing data on lifestyle and clinical factors determined for this study were excluded, resulting in 33,714 participants for analysis of the present study. A study flow chart illustrating the inclusion criteria of the study is shown in Fig. 1. This study was approved by and registered in the National Institute of Health Research and Development (NIHRD), Ministry of Health, Republic of Indonesia (Ref No. IR.03.01/8/3892/2022). Ethical approval for the 2018 RISKESDAS survey was obtained from the Health Research Ethics Committee of NIHRD (Ref No. LB.02.01/2/KE.267/2017). All respondents have signed written informed consent before participating in the survey. Our current study was exempted from ethical approval by the Health Research Ethical Committee of the Faculty of Medicine Universitas Indonesia (Ref No. ND-1/UN2.F1/ETIK/PPM.00.02/2023) as the database of the 2018 RISKESDAS is publicly available and anonymized.

### 2.2 Data collection

A set of multiple blocks of interviewer-assisted questionnaires were used to record data on socio-demographics, history of diseases, and lifestyle determinants. The lifestyle factors examined in the survey include physical activity, fruit and vegetable consumption, smoking, alcohol intake, consumption of high-risk foods, and hygiene-related behaviours. Anthropometry, blood pressure, and blood glucose measurements were performed by the enumerators on the survey date. Additionally, the clinical chemistry measurements (lipid profiles and creatinine levels) using participants’ venous blood samples were pooled and measured in the standardized national laboratory of NIHRD. The variables used in this study were measured as described below.

#### 2.2.1 Socio-demographic determinants

Age, sex, marital status, level of education, employment status, and type of employment were obtained using standardized questionnaires. The level of education was categorized as low (no formal education after primary school), intermediate (high school), and high (college/university). The type of employment was categorized as currently in education, unemployed/retired, working in the formal sector (civil servant, army, police, private employee, entrepreneur), and working in the informal sector (farmer, fisherman, labor, driver, domestic helper). Socio-economic status was based on the ownership of household assets, average income, and expenditure, categorized into quintiles. A higher number represents a higher socio-economic status. The urban and rural areas were defined according to the criteria established by BPS in 2010 (Indonesian Central Bureau of Statistics, 2010), including population density/km^2^, farming household percentage, and availability/accessibility for urban-related facilities. Each criteria has a certain score and a total score ≥10 was considered an urban area, with people living in those areas considered members of the urban population (Suppl Table 1).

#### 2.2.2 Lifestyle factors

Physical activity was reported as frequency (days/week) and duration (minute/day) of moderate and vigorous activity, which were restructured to hours per week of metabolic equivalents (Wendel-Vos et al., 2003). Being physically active was defined as moderate to vigorous physical activity (MVPA) of ≥30 min/day for 5 days or ≥150 min/week (Buku Panduan GERMAS-Gerakan Masyarakat Hidup Sehat, 2018).

In the RISKESDAS questionnaire, fruit and vegetable intake was estimated with a simplified semiquantitative food frequency questionnaire as the number of portions eaten per day. In this study, the adequate intake of fruits and vegetables was defined as total consumption of ≥400 g/day or ≥5 portions/day (Buku Panduan GERMAS-Gerakan Masyarakat Hidup Sehat, 2018).

Smoking status was assessed as never, former, and current smoker. Additionally, the pack-years of smoking were also calculated. Alcohol consumption was estimated by the number of portion glasses per day, with display cards as visual aids, summed across all types of alcohol and restructured to the unit of alcohol per day.

#### 2.2.3 Clinical factors

Body weight was measured by a calibrated digital FESCO™ weight scale to the nearest 0.1 kg, and height was measured without shoes using a calibrated, vertically fixed tape. Body mass index (BMI) was calculated by dividing body weight (kg) by the square of height (m^2^) and categorized based on the WHO criteria for the Asia-Pacific population (Inoue et al., 2000). Waist circumference was measured halfway between the iliac crest and the lowest rib using a flexible tape (SECA Model 201, Seca Gmbh Co, Hamburg, Germany).

Blood pressure was obtained by a digital sphygmomanometer at the left arm and upright sitting position after 5 min rest (HEM-7200, Omron Healthcare Co, Ltd, Kyoto, Japan). The average of three measurements was used for analysis. Hypertension was defined as systolic blood pressure (SBP) ≥140 mmHg and/or diastolic blood pressure (DBP) ≥90 mmHg or previous diagnosis of hypertension with current use of anti-hypertensive medications (Unger et al., 2020). Serum total, HDL-, and LDL-cholesterol, as well as triglyceride levels were measured using standard clinical chemistry methods (Roche® enzymatic assay) (Dany et al., 2020). Based on the criteria from The Indonesian Society of Endocrinology 2021, dyslipidemia was defined as one or more of the following criteria: total cholesterol ≥200 mg/dL, LDL-cholesterol ≥130 mmHg, HDL-cholesterol <40 mmHg in men or <50 mmHg in women, and triglyceride ≥150 mg/dL (Dislipidemia, 2021).

### 2.3 Assessment of diabetes status

The definition of diabetes was based on the combination of known diabetes, i.e., a previous diagnosis of diabetes or use of anti-diabetes medication, and unknown diabetes based on blood glucose criteria according to the American Diabetes Association (ADA) 2022 guidelines, which include one or more of the following:(American Diabetes Association Professional Practice, 2022) fasting plasma glucose (FPG) ≥126 mg/dL, OR 2-hour plasma glucose (2 h-PG) ≥200 mg/dL during oral glucose tolerance test (OGTT), OR random blood glucose ≥200 mg/dL with classic symptoms of hyperglycemia or hyperglycemia crisis. In the survey, random (n = 9,137), fasting (n = 24,403), and 2-hour post-OGTT (n = 23,228) blood glucose were measured using capillary blood samples (Accu-Chek Performa, Roche Diagnostics GmbH, Mannheim, Germany). For the respondents who were not fasting on the survey date, random blood glucose was measured instead of fasting and 2-hour post-OGTT blood glucose. HbA1c was not measured during the survey.

### 2.4 Statistical analysis

Weighted analyses were performed to account for the differences in geographical density and urban/rural distribution across the 34 provinces in Indonesia. Therefore, percentages and proportions were given instead of the number of participants. Continuous variables were summarized as mean with standard deviation (SD) for normally distributed data and median (25th, 75th percentile) for non-normal distribution. Categorical variables were presented as proportions with 95 % confidence intervals (95 % CI). Additionally, the differences between urban and rural populations were presented as mean or proportion differences with 95 % CI.

We performed multivariable logistic regression analyses, stratified by urban and rural status, to calculate odds ratios (ORs) with 95 % CIs for the associations between lifestyle and clinical determinants with the total prevalence of diabetes. The associations between lifestyle factors and diabetes were adjusted for socio-demographic determinants (age, sex, education, occupation, marital status, and socio-economic status) and BMI. The associations between clinical factors and diabetes were adjusted for socio-demographic, lifestyle factors, and BMI. The lifestyle and clinical factors were both modeled as continuous and as categorical variables based on known cut-offs from previous literatures. All continuous variables were modeled based on their actual unit, except for MVPA duration and smoking pack-years, which used per standardized (SD) unit for better interpretation. For lifestyle factors, the behaviors considered as part of a healthy lifestyle based on national guideline recommendation (Buku Panduan GERMAS-Gerakan Masyarakat Hidup Sehat, 2018) will serve as reference. These include as follows: physically active, defined as ≥150 min/week (≥30 min/day for 5 days) of moderate-vigorous physical activity; adequate intake of fruits and vegetables, defined as intake of ≥5 portions/day; never smoker; and no alcohol consumption.

To examine the differences between the populations within one analysis, we generated new categorical variables for the combinations of each risk factor and the population, using the non-exposed (‘healthy’) urban population as the reference. All analyses were performed using STATA (version 16.0, StataCorp, College Station, TX, USA).

## 3 Results

### 3.1 Socio-demographic, lifestyle, and clinical factors in Indonesian urban and rural populations

More participants in the urban population had a higher education [proportion difference (95 % confidence interval/CI): 5.5 % (4.8; 6.3)] and belonged to the highest socio-economic quintile [23.4 % (21.4; 25.3)] compared to rural population. In terms of lifestyle factors, rural population was more physically active [73,5 % in urban vs. 85,3 % in rural, differences −11.8 % (−13.5; −0.1)], more often had an adequate fruit and vegetable intake [3,4 % vs. 4,2 % in urban and rural, respectively, differences −0.8 % (−1.5; −0.1)], and had a higher prevalence of current smokers, than the urban population (Table 1).

**Table 1:** Differences in socio-demographic characteristics, lifestyle factors, and clinical factors between Indonesian urban (n = 17,129) and rural (n = 16,585) populations.

	Urban	Rural	Differences^15^
	(55 %)	(45 %)	(95 CI)
**Socio-demographic**			
Age, years	42.6 (14.9)	44.5 (16.7)	−1.8 (−2.2; −1.4)
Sex (% men)	50.3 (49.6; 51.0)	50.5 (48.8; 50.1)	−0.2 (−1.1; 0.7)
Level of education (% high^1^)	8.2 (7.5; 8.9)	2.6 (2.3; 3.0)	5.5 (4.8; 6.3)
Type of employment (% informal sector^2^)	26.4 (25.2; 27.5)	53.0 (51.7; 54.2)	−26.7 (−28.3; −24.9)
Marital status (% married)	73.8 (73.0; 74.6)	79.1 (78.3; 79.8)	−5.3 (−6.4; −4.2)
Socio-economic status (% highest/5th quintile)	32.9 (31.2; 34.7)	9.5 (8.7; 10.4)	23.4 (21.4; 25.3)

**Lifestyle Factors**			
Physically active^3^ (%)	73.5 (72.1; 74.9)	85.3 (84.2; 86.3)	−11.8 (−13.5; −0.10)
Moderate-vigorous physical activity duration* (hours/week)	11.5 (2; 28)	21 (7; 42)	−8.1 (−9.1; −7.1)
Adequate fruit and vegetable intake^4^ (%)	3.4 (3.0; 3.8)	4.2 (3.6; 4.9)	−0.8 (−1.5; −0.1)
Fruit and vegetable intake* (portion/day)	1.4 (0.9; 2.1)	1.4 (1.0; 2.6)	−0.2 (−0.2; −0.1)
Smoking behaviour (% current smoker)	31.8 (30.9; 32.7)	37.1 (36.2; 37.9)	−5.3 (−6.5; −4.0)
Pack years*^5^	10.1 (4.2; 19.2)	12 (5.8; 21.5)	−1.7 (−2.5; −0.9)
Alcohol consumption (% current drinker)	2.2 (2.0; 2.5)	1.7 (1.5; 2.0)	0.5 (0.1; 0.9)
Quantity*^6^ (unit alcohol/day)	0.2 (0.1; 1.0)	0.3 (0.1; 1.5)	0.2 (−0.5; 1.0)

**Clinical Factors**			
BMI (kg/m^2^)	24.4 (4.7)	23.1 (4.6)	1.3 (1.2; 1.4)
BMI categories^7^ (%)			
Underweight (<18.5 kg/m^2^)	9.3 (8.8; 9.9)	11.9 (11.3; 12.6)	−2.6 (−3.4; −1.7)
Normo-weight (18.5–22.9 kg/m^2^)	33.1 (32.2; 34.0)	44.1 (43.2; 45.1)	−11.1 (−12.3; −9.8)
Overweight (23.0–24.9 kg/m^2^)	16.8 (16.2; 17.4)	15.0 (14.4; 15.6)	1.8 (1.0; 2.7)
Obesity (≥25.0 kg/m^2^)	40.7 (39.8; 41.7)	28.9 (28.0; 29.8)	12.8 (11.4; 14.1)
Waist circumference, cm	81.8 (11.8)	77.7 (12.2)	4.1 (3.7; 4.5)
Men	81.3 (10.8)	76.3 (10.3)	5.0 (4.5; 5.6)
Women	82.3 (12.7)	79.2 (13.9)	3.1 (2.6; 3.6)
Abdominal obesity^8^ (%)	41.2 (40.1; 42.2)	28.4 (27.5; 29.3)	12.8 (11.4; 14.1)
Men	24.5 (23.2; 25.7)	10.4 (9.6; 11.3)	14.0 (12.5; 15.5)
Women	58.1 (56.8; 59.3)	46.7 (45.4; 48.0)	11.4 (9.5; 13.2)
Systolic blood pressure, mmHg	131.3 (23.0)	132.7 (25.0)	−1.4 (−2.1; −0.8)
Diastolic blood pressure, mmHg	84.6 (12.4)	83.9 (13.2)	0.7 (0.3; 1.1)
Hypertension^9^ (%)	40.2 (39.2; 41.1)	39.3 (38.3; 40.3)	0.9 (−0.5; 2.2)
Total cholesterol, mmol/L	4.7 (1.0)	4.6 (1.1)	0.1 (0.1; 0.1)
Hypercholesterolemia^10^ (%)	29.9 (29.0; 30.8)	26.4 (25.5; 27.3)	3.5 (2.2; 4.7)
LDL-cholesterol, mmol/L	3.2 (0.8)	3.1 (0.9)	0.1 (0.1; 0.1)
High LDL-cholesterol^11^ (%)	38.7 (37.7; 39.7)	35.0 (34.0; 36.0)	3.7 (2.2; 5.1)
HDL-cholesterol, mmol/L	1.2 (0.3)	1.2 (0.3)	0.0 (−0.0; 0.0)
Low HDL-cholesterol^12^ (%)	40.6 (39.6; 41–6)	41.2 (40.2; 42.2)	−0.6 (−2.0; 0.9)
Triglyceride, mmol/L	1.5 (1.1)	1.4 (1.0)	0.1 (0.1; 0.1)
Hypertriglyceridemia^13^ (%)	28.4 (27.5; 29.2)	25.7 (24.8; 26.5)	2.7 (1.5; 3.9)
Dyslipidemia^14^ (%)	69.5 (68.7; 70.4)	68.0 (67.1; 68.9)	1.5 (0.3; 2.8)
Random blood glucose, mmol/L	6.2 (2.4)	6.1 (2.4)	0.1 (−0.0; 0.2)
Fasting blood glucose, mmol/L	5.7 (1.8)	5.6 (1.6)	0.1 (0.1; 0.2)
2-hour glucose post OGTT, mmol/L	8.1 (2.9)	8.1 (2.9)	−0.0 (−0.2; 0.1)

Data were presented as mean (SD) for normally distributed continuous variables and median (25th–75th percentiles) for not-normally distributed continuous variables. Categorical variables were presented as percentage (95% confidence interval). Results were based on analyses weighted towards geographical density and urban–rural distribution in Indonesia.

*not-normally distributed continuous variables.

1 High education level includes participants who currently studying or having degree in college or university.

2 Informal sector employment includes farmer, fisherman, labor, driver, and domestic helper.

3 Physically active was defined as moderate to vigorous physical activity of ≥30 min/day for 5 days or ≥150 min/week.

4 Adequate fruit and vegetable intake was defined as total intake of fruit and vegetable ≥5 portions/day.

5 calculated from individuals who smoke.

6 calculated from individuals who drink alcohol.

7 BMI categories were based on the WHO cut-offs for Asian population.

8 Ethnic-Specific (Asian) waist-circumference cut-offs for abdominal obesity were ≥90 cm for men and ≥80 cm for women.

9 Hypertension was defined as systolic blood pressure ≥140 mmHg AND/OR diastolic blood pressure ≥90 mmHg OR previous hypertension diagnosis with current use of anti-hypertensive medications.

10 Hypercholesterolemia was defined as total cholesterol levels ≥5.2 mmol/L ((≥200 mg/dL).

11 High LDL-cholesterol was defined as LDL-cholesterol levels ≥3.4 mmol/L (≥130 mg/dL).

12 Low HDL-cholesterol was defined as HDL-cholesterol levels <1.0 mmol/L (<40 mg/dL) in men or <1.3 mmol/L (<50 mg/dL) in women.

13 Hypertriglyceridemia was defined as triglyceride levels ≥1.7 mmol/L (≥150 mg/dL).

14 Dyslipidemia was defined based of one or more of the following criteria: total cholesterol ≥200 mg/dL, OR LDL-cholesterol ≥130 mg/dL, OR low triglyceride (≥150 mg/dL), OR low HDL-cholesterol (<40 mg/dL in men or <50 mg/dL in women).

15 Differences were calculated as values in urban minus values in rural. For not normally distributed continuous variables, the differences were calculated using mean and standard error to obtain the mean differences and its 95 % confidence intervals.

BMI and waist circumference were higher in urban than rural population. Moreover, the prevalence of obesity, whether classified by BMI categories (40.7 % vs. 28.9 %, for urban and rural, respectively) or abdominal obesity criteria (41.2 % for urban and 28.4 % for rural), was also higher in the urban population. Systolic blood pressure was higher in rural compared to urban population, and the opposite was observed for DBP, resulting in similar rates of hypertension. No differences were observed for total cholesterol, LDL-cholesterol, HDL-cholesterol, and triglyceride levels between the two populations. However, urban participants showed higher proportions of hypercholesterolemia, high LDL-cholesterol, hypertriglyceridemia, and dyslipidemia (Table 1).

### 3.2 Diabetes prevalence in the Indonesian urban and rural populations

**Fig. 2:**
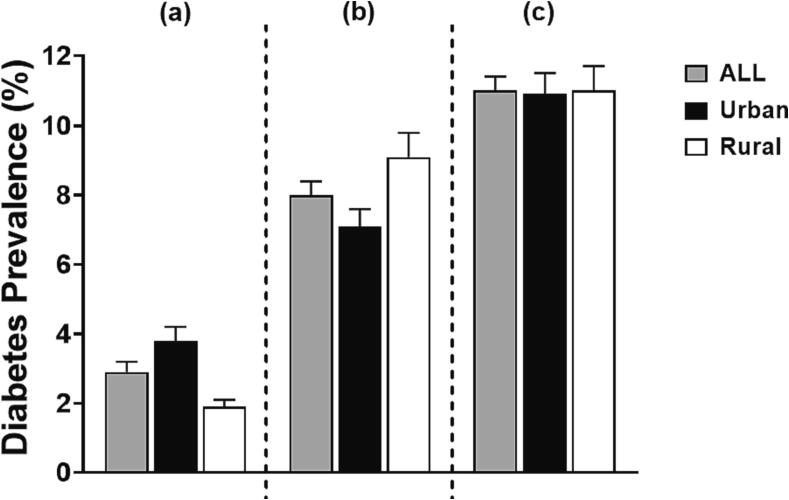
The prevalence of diabetes between Indonesian urban and rural population, (a). Known (previously diagnosed and treated) diabetes; (b). Unknown (undiagnosed and untreated) diabetes; (c). Total prevalence*. *The combination of prevalence of known diabetes and unknown diabetes using blood glucose criteria as follows: fasting plasma glucose (FPG) ≥126 mg/dL or 7 mmol/L, OR 2-hour plasma glucose (2 h-PG) ≥200 mg/dL or 11.1 mmol/L after an oral glucose tolerance test (OGTT), OR random blood glucose ≥200 mg/dL or 11.1 mmol/L with classic symptoms of hyperglycemia or hyperglycemia crisis.

There were no differences in the total prevalence of diabetes between Indonesian urban and rural populations [proportion (95 %CI): 10.9 % (10.4; 11.5) and 11.0 % (10.4; 11.7) for urban and rural, respectively]. Nevertheless, the proportion of individuals with known diabetes was twice as high in the urban population [3.8 % (3.5; 4.2)] than in the rural population [1.9 % (1.6; 2.1]. This resulted in a relatively high prevalence of undiagnosed and untreated diabetes, especially in the rural population [7.1 % (6.7; 7.6) in urban and 9.1 % (8.6; 9.8) in rural population] (Fig. 2).

### 3.3 Lifestyle factors and prevalent diabetes in Indonesian urban and rural populations

A longer duration of MVPA was associated with lower risk of prevalent diabetes [prevalence odds ratio (95 % confidence interval): 0.91 (0.85; 0.98) for urban and [0.94 (0.89; 1.00) for rural population] (Table 2). The results were similar when using categorical variables in the models, showing a higher risk of prevalent diabetes with physical inactivity, especially in the urban population (Suppl. Table 2). In contrast with majority of previous findings, we found a positive correlation between fruit and vegetable intake with prevalent diabetes in the urban population (Table 2). Although, sensitivity analysis suggested potential confounding by sex, age, and BMI (Suppl. Tables 3 and 4). Additionally, urban population displayed inverse associations between smoking pack-years and alcohol consumption with the prevalence of diabetes (Table 2). Moreover, when compared to the non-smoker group, current smoker was inversely associated with prevalent diabetes in both populations (Suppl. Table 2). Further sensitivity analysis indicated that current smokers has a lower BMI and a higher proportion of men than non-smokers (Suppl. Tables 5 and 6).

**Table 2:** Association of lifestyle factors as continuous variables with prevalent diabetes in Indonesian urban and rural populations.

Variables	Model^3^	Prevalence Odds Ratio (95 %CI)	
		Urban	Rural
Moderate/vigorous physical activity, per 1 SD = 21.4 h/week	Crude OR	0.86 (0.81; 0.92)	0.90 (0.85; 0.95)
	Model 1	0.92 (0.87; 0.99)	0.97 (0.91; 1.02)
	Model 2	0.92 (0.86; 0.98)	0.96 (0.90; 1.02)
	Model 3	0.91 (0.85; 0.98)	0.94 (0.89; 1.00)

Fruit and vegetable intake (portion/day)	Crude OR	1.11 (1.07; 1.14)	1.01 (0.97; 1.05)
	Model 1	1.08 (1.04; 1.12)	1.01 (0.97; 1.05)
	Model 2	1.07 (1.03; 1.11)	1.02 (0.97; 1.06)
	Model 3	1.06 (1.02; 1.11)	1.01 (0.96; 1.05)

Smoking,^1^ per 1 SD = 15.2 pack-years	Crude OR	1.35 (1.23; 1.49)	1.26 (1.15; 1.37)
	Model 1	0.97 (0.85; 1.11)	1.05 (0.93; 1.19)
	Model 2	0.94 (0.81; 1.10)	1.01 (0.88; 1.17)
	Model 3	0.94 (0.81; 1.10)	1.00 (0.86; 1.16)

Alcohol consumption^2^ (unit alcohol/day)	Crude OR	0.87 (0.78; 0.96)	1.01 (0.91; 1.11)
	Model 1	0.85 (0.78; 0.93)	1.02 (0.95; 1.10)
	Model 2	0.86 (0.77; 0.96)	1.02 (0.95; 1.10)
	Model 3	0.86 (0.76; 0.96)	1.02 (0.95; 1.10)

Data were presented as prevalence odds ratio (OR) and its 95% confidence interval (CI).

Model 1: adjusted for age and sex.

Model 2: adjusted for model 1 + other socio-demographic determinants (education, employment, marital, and socio-economic status).

Model 3: adjusted for model 2 + body mass index.

SD: standardized unit.

1 calculated from individuals who smoke.

2 calculated from individuals who drink alcohol.

3 Model for adjustment:

### 3.4 Clinical factors and prevalent diabetes in Indonesian urban and rural populations

All clinical factors, either modelled as continuous (Table 3) or as categorical variables (Suppl. Table 7), were associated with prevalent diabetes both in urban and rural populations.

**Table 3:** Association of clinical factors as continuous variables with prevalent diabetes in Indonesian urban and rural populations.

Variables	Model^1^	Prevalence Odds Ratio (95 %CI)	
		Urban	Rural
BMI (kg/m^2^)	Crude OR	1.06 (1.05; 1.07)	1.06 (1.05; 1.07)
	Model 1	1.06 (1.05; 1.07)	1.06 (1.05; 1.08)
	Model 2	1.06 (1.05; 1.07)	1.06 (1.05; 1.08)
	Model 3	1.06 (1.05; 1.07)	1.06 (1.05; 1.08)

Waist circumference (cm), per 5 unit increase	Crude OR	1.19 (1.16; 1.21)	1.15 (1.12; 1.18)
	Model 1	1.15 (1.13; 1.18)	1.14 (1.11; 1.17)
	Model 2	1.15 (1.12; 1.17)	1.14 (1.11; 1.17)
	Model 3	1.14 (1.12; 1.17)	1.14 (1.11; 1.16)
	Model 4	1.11 (1.07; 1.15)	1.11 (1.07; 1.15)

Systolic blood pressure (mmHg), per 10 unit increase	Crude OR	1.25 (1.23; 1.27)	1.20 (1.17; 1.22)
	Model 1	1.12 (1.10; 1.15)	1.10 (1.08; 1.13)
	Model 2	1.12 (1.10; 1.15)	1.10 (1.07; 1.13)
	Model 3	1.12 (1.10; 1.15)	1.09 (1.07; 1.12)
	Model 4	1.10 (1.08; 1.13)	1.07 (1.05; 1.10)

Diastolic blood pressure (mmHg), per 5 unit increase	Crude OR	1.15 (1.12; 1.17)	1.15 (1.13; 1.18)
	Model 1	1.09 (1.07; 1.12)	1.11 (1.09; 1.13)
	Model 2	1.10 (1.08; 1.12)	1.10 (1.08; 1.13)
	Model 3	1.10 (1.07; 1.12)	1.10 (1.08; 1.12)
	Model 4	1.07 (1.05; 1.09)	1.07 (1.05; 1.10)

Total cholesterol (mmol/L)	Crude OR	1.67 (1.58; 1.75)	1.49 (1.42; 1.58)
	Model 1	1.40 (1.33; 1.48)	1.30 (1.23; 1.38)
	Model 2	1.40 (1.32; 1.49)	1.30 (1.22; 1.37)
	Model 3	1.40 (1.33; 1.49)	1.29 (1.22; 1.37)
	Model 4	1.37 (1.29; 1.45)	1.25 (1.18; 1.32)

LDL cholesterol (mmol/L)	Crude OR	1.66 (1.56; 1.76)	1.49 (1.40; 1.58)
	Model 1	1.40 (1.32; 1.49)	1.30 (1.22; 1.39)
	Model 2	1.39 (1.30; 1.48)	1.30 (1.22; 1.39)
	Model 3	1.39 (1.29; 1.48)	1.30 (1.21; 1.38)
	Model 4	1.34 (1.25; 1.43)	1.23 (1.15; 1.32)

HDL cholesterol (mmol/L)	Crude OR	0.65 (0.54; 0.78)	0.82 (0.67; 0.99)
	Model 1	0.35 (0.29; 0.43)	0.47 (0.38; 0.58)
	Model 2	0.36 (0.29; 0.44)	0.46 (0.37; 0.57)
	Model 3	0.34 (0.28; 0.42)	0.45 (0.36; 0.55)
	Model 4	0.40 (0.32; 0.50)	0.52 (0.42; 0.65)

Triglyceride (mmol/L)	Crude OR	1.32 (1.25; 1.40)	1.32 (1.24; 1.41)
	Model 1	1.30 (1.22; 1.39)	1.32 (1.24; 1.40)
	Model 2	1.31 (1.23; 1.41)	1.32 (1.24; 1.41)
	Model 3	1.33 (1.24; 1.42)	1.33 (1.25; 1.42)
	Model 4	1.28 (1.20; 1.37)	1.28 (1.20; 1.36)

Model 1: adjusted for age and sex.

Model 2: adjusted for model 1 + other socio-demographic determinants (education, employment, marital, and socio-economic status).

Model 3: adjusted for model 2 + lifestyle determinants (physical activity, fruit and vegetable intake, smoking behaviour, and alcohol consumption).

Model 4: adjusted for model 3 + body mass index.

Data were presented as prevalence odds ratio (OR) and its 95 % confidence interval (CI).

1 Model for adjustment.

### 3.5 The differences in the association of lifestyle and clinical factors with diabetes prevalence between Indonesian urban and rural populations

**Fig. 3:**
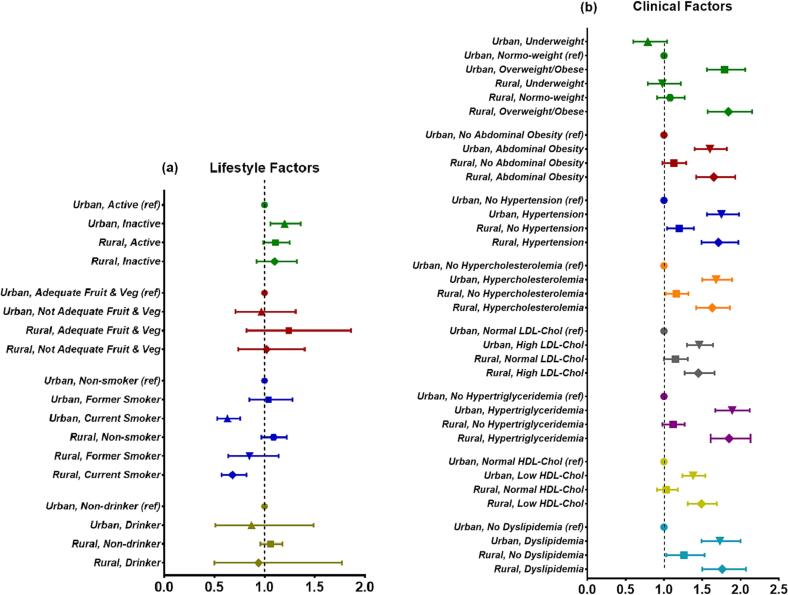
The differences in the association of lifestyle (a) and clinical (b) factors with prevalent diabetes between Indonesian urban and rural populations. Data were presented as prevalence odds ratio (OR) with its 95 % confidence interval (95 %CI) compared with the reference category (ref), i.e., urban population without risk factors. For models in (a), associations were adjusted for age, sex, socio-demographic determinants (level of education, type of employment, marital status, and socio-economic status), and body mass index (BMI). For models in (b), associations were adjusted for age, sex, socio-demographic determinants (level of education, type of employment, marital status, and socio-economic status), lifestyle factors (moderate/vigorous physical activity, fruit and vegetable intake, smoking, and alcohol consumption), and BMI. Inactive was defined as moderate/vigorous physical activity <150 min/week. Not adequate fruit and vegetable intake was defined as fruit and vegetable consumption <5 portions/day. BMI categories were based on the WHO cut-offs for Asian population: underweight (BMI <18.5 kg/m^2^), normo-weight (BMI 18.5–22.9 kg/m^2^), and overweight/obese (BMI ≥23.0 kg/m^2^). Ethnic-Specific (Asian) waist-circumference cut-offs for abdominal obesity were >90 cm for men and >80 cm for women. Hypertension was defined as systolic blood pressure >140 mmHg AND/OR diastolic blood pressure >90 mmHg OR previous hypertension diagnosis with current use of anti-hypertensive medications. Hypercholesterolemia was defined as total cholesterol levels ≥5.2 mmol/L ((≥200 mg/dL). High LDL-cholesterol was defined as LDL-cholesterol levels ≥3.4 mmol/L (≥130 mg/dL). Low HDL-cholesetrol was defined as HDL-cholesterol levels <1.0 mmol/L (<40 mg/dL) in men or <1.3 mmol/L (<50 mg/dL) in women. Hypertriglyceridemia was defined as triglyceride levels ≥1.7 mmol/L (≥150 mg/dL). Dyslipidemia was defined based of one or more of the following criteria: total cholesterol ≥200 mg/dL, OR LDL-cholesterol ≥130 mg/dL, OR low triglyceride (≥150 mg/dL), OR low HDL-cholesterol (<40 mg/dL in men or <50 mg/dL in women).

In comparison with the urban-physically active group, a higher prevalence odds ratio of diabetes was seen for urban-inactive [prevalence OR (95 %CI): 1.17 (1.03; 1.33)], but not for the rural-inactive group [0.97 (0.81; 1.16)]. No differences were observed between urban and rural populations with inadequate fruit and vegetable intake compared to the urban-adequate group. There were also no differences between urban and rural current smokers in comparison with the urban reference group (Fig. 3a).

With regard to clinical factors, both urban and rural populations with overweight or obesity exhibited higher prevalence ORs than the urban-normo-weight reference group, although there were no differences between the two groups [1.79 (1.56; 2.06) vs. 1.84 (1.57; 2.15) for urban-overweight/obese and rural-overweight/obese, respectively]. Similar patterns were observed for all other clinical factors, showing no differences in the prevalence ORs of diabetes between the urban and rural populations with clinical risk factors compared to the urban population without risk factors. Additionally, in Fig. 3b, it’s evident that the rural population without lifestyle or clinical risk factors had a higher risk of prevalent diabetes compared to the urban population without risk factors. Additional analyses revealed that this rural population without risk factors was somewhat older than the urban reference group (Suppl. Table 8).

## 4 Discussion

In this study, we observed several differences in lifestyle and clinical determinants between Indonesian urban and rural population aged ≥15 years old. The rural population showed a better profile in lifestyle and clinical factors compared to the urban population. Although the total prevalence of diabetes was similar between the two populations, a higher prevalence of known diabetes was observed in the urban than in the rural population. In terms of lifestyle, physical inactivity was a risk factor for diabetes, particularly in the urban setting. Whereas overweight/obesity, abdominal obesity, hypertension, and dyslipidemia were all risk factors for diabetes in both populations, there were no differences in the relation between these risk factors and the prevalence of diabetes between urban and rural populations.

The observed higher physical activity levels in rural compared to urban population aligned with previous study (Machado-Rodrigues et al., 2014). This finding could be attributed by the greater proportion of rural individuals engaged in physically demanding informal sector work, as demonstrated in our earlier research (Sigit et al., 2022). Furthermore, our study affirmed an inverse association between longer duration of MVPA per week with diabetes, and similarly, physical inactivity is associated with higher risk of diabetes, which were more pronounced in the urban population. This could be explained by previous findings that leisure time, but not occupational physical activity, is associated with a reduced risk of diabetes (Medina et al., 2018).

In contrast to previous study (Halvorsen et al., 2021), we observed a positive association between fruit and vegetable consumption and diabetes in the urban population. One potential explanation for this finding could be due to reverse causation, individuals with diabetes might have altered their diet after the diagnosis of diabetes. Additionally, in our urban population, factors like sex, age, and BMI emerged as strong confounders for this association. The highest tertile of fruit and vegetable intake correlated with more women, older age, and higher BMI compared to the lowest tertile. Notably, the types of fruit or vegetable and serving methods were not evaluated in this study. Previous study showed that certain types fruits or vegetables, and juices were positively associated with diabetes (Barouti et al., 2022).

The finding of an inverse association between current smokers with diabetes was also reported in several previous studies to be confounded by sex and BMI (Liu et al., 2017, Wang et al., 2019). Indeed, our study revealed that the current smokers had a lower BMI and male predominance than the non-smokers. Although, adjustment for sex and BMI did not fully attenuate the associations. The observed inverse association between alcohol consumption and diabetes in the urban population supports prior finding suggesting light/moderate drinking might lower the risk of diabetes (Holst et al., 2017). However, this finding should be interpreted carefully due to potential residual confounding, as current drinkers may represent a small selective group of the Indonesian urban population who drink alcohol, and may have a lower risk of diabetes because of other reasons than alcohol consumption.

The higher BMI, waist circumference, and obesity prevalence in urban compared to rural population observed in the current study, confirmed our previous findings (Kurniawan et al., 2022). This present study also found these adiposity indices and obesity are positively correlated with diabetes in both populations, similar to what had been observed previously (Bellou et al., 2018). Additionally, our study confirmed higher blood pressure and hypertension status as well-established clinical risk factors for diabetes (Kim et al., 2015), for both urban and rural populations. Consistent with prior study (de Groot et al., 2019), we reported a higher prevalence of hypercholesterolemia, high LDL-cholesterol, hypertriglyceridemia, and dyslipidemia in the urban than rural population. Correspondingly, we also observed positive associations between these lipid abnormalities and prevalent diabetes, in concordance with earlier findings (Peng et al., 2021).

Despite rural population showing a better lifestyle and clinical profiles than the urban population, there were no differences in the associations of these risk factors with prevalent diabetes between the two populations, except for physical activity. Nevertheless, it must be noted there is an alarming increase of BMI in the rural areas of LMICs, possibly due to transition from undernutrition to complex malnutrition with over consumption of low-quality calories (Bixby et al., 2019), which may lead to increased future rates of diabetes in rural populations. Our previous studies also support this postulate, showing more unfavorable metabolic changes in rural compared to urban subjects, when exposed towards short-term high-fat high-calories diet intervention (Tahapary et al., 2018), as well as a relatively long-term urban lifestyle (Kurniawan et al., 2022).

We observed no differences in the overall diabetes prevalence between urban and rural population. This supports the finding from The 2014 Indonesia Family Life Survey (IFLS) which showed a similar pattern (7.5 % in urban vs. 6.8 % in rural population) using HbA1c measurement (Mulyanto et al., 2019). Interestingly, another study utilizing the same IFLS database reported a twice higher prevalence of known diabetes in the Indonesian urban compared to rural population (2.9 % vs. 1.4 %, for urban and rural, respectively) (Indrahadi et al., 2021), similar with our current findings.

Based on the findings from our current study and the 2014 IFLS database, it’s evident that majority of individuals with diabetes in Indonesia were undiagnosed and untreated, particularly in the rural population. This may be attributed to several challenges faced in rural areas, such as limited healthcare access (Kosen et al., 2014), socio-economic constraints prioritizing other household needs (Mulyanto et al., 2019), and lower education levels leading to a lack of awareness about diabetes screening importance (Asril et al., 2019). Moreover, this prevalence of undiagnosed diabetes in Indonesia surpass the global average of 44 % reported by IDF in 2021. Strikingly, there has been no improvement in the last decade, with the 2007 Indonesian Basic Health Survey indicating approximately 74 % of the 5.7 % Indonesian population with diabetes being undiagnosed (Riset Kesehatan Dasar (RISKESDAS), 2007). Thus, urgent actions by all related stakeholders are needed to address this situation, since diabetes leads to many health complications, even worse if left untreated or sub-optimally managed (Ali et al., 2020, Bain et al., 2020), imposing an even higher burden on the Indonesian health and economic system.

Our study, with relatively large number of participants and nationally representative data could provide robust insights potentially generalized to the entire Indonesian population. Another added point offered by this study is the attempt to evaluate the magnitude of differences between urban and rural population on the association of lifestyle and clinical factors with prevalent diabetes. However, some limitations still need to be considered. First, the unavailability of HbA1c data might lead to an underestimation of the total prevalence of diabetes. Second, the observational and cross-sectional design of this study hinders evaluating temporal relationship and may introduce reverse causation and residual confounding that might explain the unexpected associations between certain lifestyle factors with diabetes. Third, the possibility of information bias, including social desirability bias, and possible measurement error, could not be fully excluded in this study. Fourth, the unavailability of lipid lowering agent usage data might cause an underestimation of the prevalence of dyslipidemia/lipid-associated disorders. Lastly, there are other factors that might differ characteristically and in the association with diabetes between rural and urban populations but not included in this study, such as consumption of high-risk foods (Neuenschwander et al., 2019), macronutrients intake (Appuhamy et al., 2014), pollution (Song et al., 2016), parasitic infection (Rennie et al., 2021), and psychological stress (Merabet et al., 2022).

## 5 Conclusion

Our study highlights a more favorable lifestyle and clinical factors in the Indonesian rural compared to urban population. Despite no differences in the total prevalence of diabetes between the two populations, a significant proportion of undiagnosed and untreated diabetes was observed, especially in the rural population. While encouraging physical activity is crucial, it's noteworthy that clinical risk factors were uniformly associated with a higher diabetes prevalence in both populations. All these findings emphasize the need for comprehensive interventions, along with supportive government health policies, to overcome the diabetes pandemic in the Indonesian population.

## Funding

The study was supported by the grant PUTI Universitas Indonesia (Grant No. NKB-762/UN2.RST/HKP.05.02/2020) and PUPT Kemenristekdikti Indonesia (Grant No. NKB-125/UN2.RST/HKP.05.00/2021). The doctoral study of the first author was funded by a scholarship from The Indonesian Endowment Fund for Education (Lembaga Pengelola Dana Pendidikan/LPDP) Ministry of Finance Republic of Indonesia, Ref S-364/LPDP.3/2019. The funders had no role in the study design, analysis, decision to publish, or preparation of the manuscript.

## CRediT authorship contribution statement

**Farid Kurniawan:** Conceptualization, Data curation, Formal analysis, Investigation, Methodology, Visualization, Writing – original draft. **Fathimah S. Sigit:** Conceptualization, Formal analysis, Methodology, Writing – review & editing. **Stella Trompet:** Methodology, Supervision, Writing – review & editing. **Em Yunir:** Funding acquisition, Writing – review & editing. **Tri Juli E. Tarigan:** Funding acquisition, Writing – review & editing. **Dante S. Harbuwono:** Funding acquisition, Writing – review & editing. **Pradana Soewondo:** Funding acquisition, Supervision, Writing – review & editing. **Dicky L. Tahapary:** Funding acquisition, Methodology, Supervision, Writing – review & editing. **Renée de Mutsert:** Conceptualization, Methodology, Software, Supervision, Writing – review & editing.

## Declaration of competing interest

The authors declare that they have no known competing financial interests or personal relationships that could have appeared to influence the work reported in this paper.

## Data Availability

The authors do not have permission to share data.
