# Supplementary material for: Lifestyle and clinical risk factors in relation with the prevalence of diabetes in the Indonesian urban and rural populations: The 2018 Indonesian Basic Health Survey
Source: Prev Med Rep. 2024 Jan 24;38:102629. doi: 10.1016/j.pmedr.2024.102629 (PMC10874845; doi:10.1016/j.pmedr.2024.102629)
Supplement: Supplementary data 1 [file mmc1.docx]

**SUPPLEMENTARY MATERIALS**


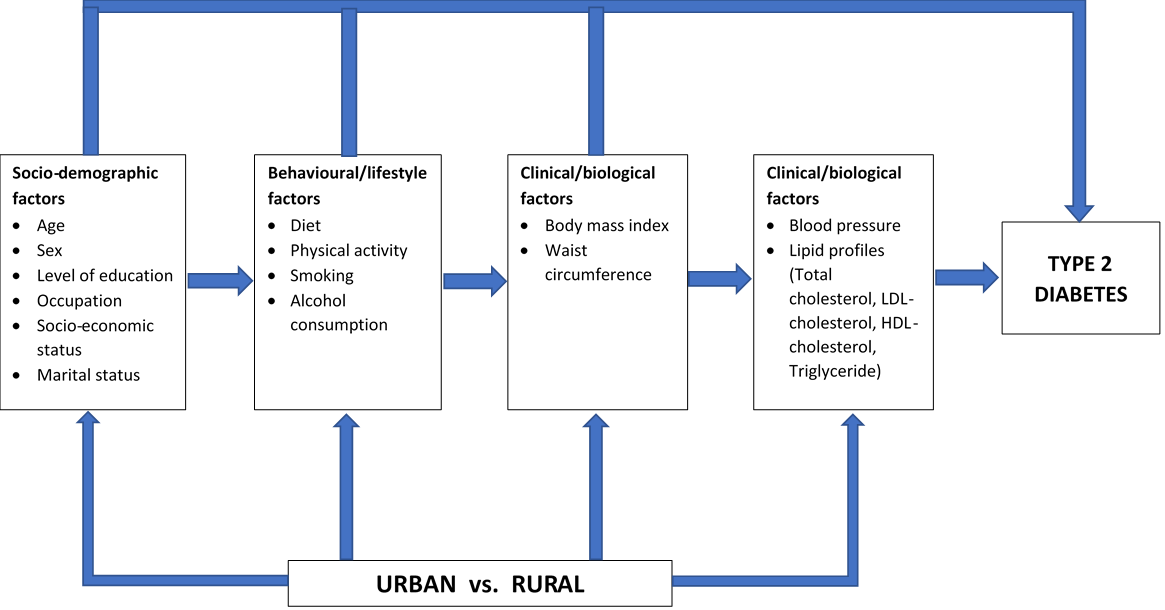


**Supplementary Figure 1. Conceptual framework and hypothesis diagram of the association between socio-demographic, lifestyle, and clinical factors with diabetes in the urban and rural populations**

**Supplementary Table 1. The 2010 Indonesian Central Bureau Statistics criteria for defining urban and rural area in Indonesia.**

| **Criteria** | | | | **Availability/accessibility for urban-related facilities** | | |
| --- | --- | --- | --- | --- | --- | --- |
| **Population density/km^2^** | **Score** | **Farming household percentage** | **Score** | **Urban-related facilities** | **Criteria** | **Score** |
| <500 | 1 | >70.00 | 1 | a. Kindergarten | - Yes OR <2.5 km - >2.5 km | 1  0 |
| 500 – 1249 | 2 | 50.00 – 69.99 | 2 | b. Junior high school |  |  |
| 1250 – 2499 | 3 | 30.00 – 49.99 | 3 | c. Senior high school |  |  |
| 2500 – 3999 | 4 | 20.00 – 29.99 | 4 | d. Market | - Yes OR <2.0 km - >2.0 km | 1  0 |
| 4000 – 5999 | 5 | 15.00 – 19.99 | 5 | e. Shops |  |  |
| 6000 – 7499 | 6 | 10.00 – 14.99 | 6 | f. Movie theatre | - Yes OR <5.0 km - 5.0 km | 1  0 |
| 7500 – 8499 | 7 | 5.00 – 9.99 | 7 | g. Hospital |  |  |
| > 8500 | 8 | <5.00 | 8 | h. Hotel/Pool/Nightclub/  Massage parlors/Salon | - Yes - No | 1  0 |
|  |  |  |  | i. Percentage of house-hold with telephone | - ≥8.00 - <8.00 | 1  0 |
|  |  |  |  | j. Percentage of house-hold with electricity | - ≥90.00 - <9000 | 1  0 |

Total score ≥10 was categorized as urban area.

**Supplementary Table 2. Association of lifestyle factors as categorical variables with prevalent diabetes in Indonesian urban and rural population.**

| **Variables** | **Urban** | | | | **Rural** | | | |
| --- | --- | --- | --- | --- | --- | --- | --- | --- |
|  | **Crude OR** | **Model 1^1^** | **Model 2^2^** | **Model 3^3^** | **Crude OR** | **Model 1^1^** | **Model 2^2^** | **Model 3^3^** |
| Moderate/vigorous physical activity  - Active  - Inactive | 1  1.24  (1.11; 1.40) | 1  1.13  (1.00; 1.28) | 1  1.12  (0.99; 1.28) | 1  1.15  (1.01; 1.31) | 1  1.10  (0.95; 1.28) | 1  0.98  (0.84; 1.14) | 1  1.00  (0.85; 1.18) | 1  1.05  0.89; 1.24) |
| Fruit and vegetable intake  - Adequate  - Not adequate | 1  0.82  (0.62; 1.07) | 1  0.94  (0.71; 1.26) | 1  1.00  (0.73; 1.36) | 1  1.00  (0.73; 1.36) | 1  0.85  (0.66; 1.08) | 1  0.81  (0.63; 1.05) | 1  0.79  (0.60; 1.04) | 1  0.82  (0.62; 1.07) |
| Smoking  - Never smoke  - Former smoker  - Current smoker | 1  1.26  (1.06; 1.49)  0.52  (0.45; 0.60) | 1  0.98  (0.80; 1.20)  0.56  (0.46; 0.67) | 1  0.96  (0.77; 1.21)  0.57  (0.47; 0.70) | 1  0.96  (0.77; 1.20)  0.61  (0.50; 0.74) | 1  0.93  (0.72; 1.20)  0.55  (0.48; 0.63) | 1  0.85  (0.64; 1.13)  0.62  (0.51; 0.76) | 1  0.89  (0.66; 1.20)  0.63  (0.51; 0.78) | 1  0.89  (0.66; 1.20)  0.68  (0.55; 0.84) |
| Alcohol consumption  - Non-drinker  - Drinker | 1  0.47  (0.29; 0.76) | 1  1.00  (0.61; 1.64) | 1  0.95  (0.55; 1.64) | 1  0.91  (0.53; 1.58) | 1  0.42  (0.24; 0.75) | 1  0.86  (0.48; 1.55) | 1  0.82  (0.44; 1.54) | 1  0.85  (0.46; 1.59) |

Data were presented as prevalence odds ratio (OR) and its 95% confidence interval (CI).

^1^Model 1: adjusted for age and sex.

^2^Model 2: adjusted for model 1 + other socio-demographic determinants (education, employment, marital, and socio-economic status)

^3^Model 3: adjusted for model 2 + body mass index

**Supplementary Table 3. Age, sex, and BMI between tertiles of fruit and vegetable intake in Indonesian urban and rural population.**

|  | **Lowest tertile** | **Mid-tertile** | **Highest tertile** |
| --- | --- | --- | --- |
|  |  | **URBAN** |  |
| Age*, years old | 41.4 (15.2) | 42.6 (14.5) | 43.9 (14.7) |
| Sex, %male | 54.6 (53.2; 56.0) | 50.2 (48.9; 51.6) | 46.1 (44.8; 47.4) |
| BMI*, kg/m^2^ | 23.8 (4.6) | 24.5 (4.7) | 24.9 (4.8) |
| Fruit and vegetable intake^#^, portion/day | 0.6 (0.4; 0.9) | 1.4 (1.1; 1.6) | 2.7 (2.1; 3.6) |
|  |  | **RURAL** |  |
| Age*, years old | 45.2 (17.6) | 43.7 (16.5) | 44.5 (16.0) |
| Sex, %male | 52.0 (50.7; 53.4) | 49.5 (48.1; 50.9) | 50.1 (49.0; 51.2) |
| BMI*, kg/m^2^ | 22.7 (4.5) | 23.2 (4.7) | 23.4 (4.7) |
| Fruit and vegetable intake^#^, portion/day | 0.7 (0.4; 1.0) | 1.3 (1.1; 1.5) | 3 (2.3; 3.7) |

*normally distributed continuous variable, presented as mean and its standard deviation.

^#^non-normally distributed continuous variable, presented as median (25^th^, 75^th^ percentile)

BMI: body mass index

**Supplementary Table 4. Association between fruit and vegetable intake tertiles and prevalent diabetes in Indonesian urban and rural population adjusted for sex, age, and BMI.**

| **Fruit and vegetable intake tertiles** | **Crude OR** | **Adjusted for sex** | **Adjusted for age** | **Adjusted for BMI** | **Adjusted for**  **sex, age & BMI** |
| --- | --- | --- | --- | --- | --- |
|  | **URBAN** | | | | |
| Lowest tertile | 1 | 1 | 1 | 1 | 1 |
| Mid-tertile | 1.14  (0.99; 1.31) | 1.12  (0.97; 1.29) | 1.12  (0.97; 1.30) | 1.09  (0.95; 1.26) | 1.07  (0.92; 1.24) |
| Highest tertile | 1.44  (1.26; 1.65) | 1.40  (1.23; 1.60) | 1.37  (1.19; 1.57) | 1.36  (1.19; 1.55) | 1.26  (1.09; 1.45) |
|  | **RURAL** | | | | |
| Lowest tertile | 1 | 1 | 1 | 1 | 1 |
| Mid-tertile | 0.93  (0.81; 1.07) | 0.91  (0.79; 1.05) | 0.98  (0.85; 1.14) | 0.95  (0.82; 1.10) | 0.95  (0.82; 1.09) |
| Highest tertile | 0.97  (0.85; 1.11) | 0.96  (0.84; 1.10) | 1.01  (0.88; 1.16) | 0.96  (0.84; 1.10) | 0.96  (0.84; 1.10) |

Data were presented as prevalence odds ratio (OR) and its 95% confidence interval (CI).

**Supplementary Table 5. Age, sex, BMI, and pack-years between the three categories of smoking habit in Indonesian urban and rural population.**

|  | **Non-smoker** | **Former smoker** | **Current smoker** |
| --- | --- | --- | --- |
|  |  | **URBAN** |  |
| Age*, years old | 41.8 (15.9) | 49.0 (14.5) | 42.6 (12.7) |
| Sex, %male | 22.1 (21.1; 23.1) | 83.5 (81.1; 85.7) | 95.1 (94.4; 95.8) |
| BMI*, kg/m^2^ | 25.1 (5.1) | 24.6 (4.6) | 22.9 (3.7) |
| Pack-years^#^ | 0 | 14.4 (6.5; 30) | 9.6 (4.0; 18.6) |
|  |  | **RURAL** |  |
| Age*, years old | 43.1 (17.5) | 51.0 (16.5) | 45.8 (15.0) |
| Sex, %male | 18.6 (17.7; 19.6) | 87.1 (84.3; 89.5) | 96.4 (95.8; 96.9) |
| BMI*, kg/m^2^ | 24.0 (5.2) | 22.6 (4.4) | 21.7 (3.4) |
| Pack-years^#^ | 0 | 16.2 (8.4; 28.2) | 12 (5.7; 21) |

*normally distributed continuous variable, presented as mean and its standard deviation.

^#^non-normally distributed continuous variable, presented as median (25^th^, 75^th^ percentile)

BMI: body mass index

**Supplementary Table 6. Association between smoking habit and prevalent diabetes in Indonesian urban and rural population adjusted for sex, age, and BMI.**

| **Smoking categories** | **Crude OR** | **Adjusted for sex** | **Adjusted for age** | **Adjusted for BMI** | **Adjusted for**  **sex, age & BMI** |
| --- | --- | --- | --- | --- | --- |
|  | **URBAN** | | | | |
| - Non-smoker  - Former smoker  - Current smoker | 1  1.26  (1.06; 1.49)  0.52  (0.45; 0.60) | 1  1.32  (1.10; 1.60)  0.55  (0.46; 0.66) | 1  0.88  (0.74; 1.05)  0.49  (0.43; 0.57) | 1  1.30  (1.09; 1.54)  0.59  (0.51; 0.68) | 1  0.97  (0.79; 1.19)  0.60  (0.50; 0.73) |
|  | **RURAL** | | | | |
| - Non-smoker  - Former smoker  - Current smoker | 1  0.93  (0.72; 1.20)  0.55  (0.48; 0.63) | 1  1.12  (0.85; 1.50)  0.69  (0.56; 0.84) | 1  0.70  (0.54; 0.90)  0.49  (0.43; 0.56) | 1  0.99  (0.76; 1.28)  0.61  (0.54; 0.70) | 1  0.85  (0.64; 1.13)  0.67  (0.55; 0.81) |

Data were presented as prevalence odds ratio (OR) and its 95% confidence interval (CI).

**Table 5. Association of clinical factors (as categorical variables) with prevalent diabetes in Indonesian urban and rural population.**

| **Variables** | **Urban** | | | | | **Rural** | | | | |
| --- | --- | --- | --- | --- | --- | --- | --- | --- | --- | --- |
|  | **Crude OR** | **Model 1** | **Model 2** | **Model 3** | **Model 4** | **Crude OR** | **Model 1** | **Model 2** | **Model 3** | **Model 4** |
| BMI categories^1^  - Underweight (<18.5 kg/m^2^)  - Normo-weight (18.5-22.9 kg/m^2^)  - Overweight (23.0-24.9 kg/m^2^)  - Obese (≥25.0 kg/m^2^) | 0.72  (0.56; 0.95)  1  1.60  (1.35; 1.89)  2.05  (1.80; 2.34) | 0.74  (0.57; 0.97)  1  1.58  (1.33; 1.87)  1.99  (1.73; 2.29) | 0.78  (0.58; 1.03)  1  1.53  (1.27; 1.84)  1.98  (1.71; 2.30) | 0.77  (0.59; 1.03)  1  1.51  (1.25; 1.81)  1.94  (1.67; 2.25) |  | 1.10  (0.92; 1.32)  1  1.31  (1.10; 1.55)  1.82  (1.59; 2.07) | 0.94  (0.79; 1.13)  1  1.31  (1.10; 1.57)  1.80  (1.56; 2.07) | 0.95  (0.79; 1.15)  1  1.29  (1.07; 1.56)  1.80  (1.55; 2.10) | 0.96  (0.79; 1.16)  1  1.27  (1.06; 1.54)  1.77  (1.52; 2.05) |  |
| Abdominal obesity^2^  - No  - Yes | 1  2.46  (2.22; 2.73) | 1  2.09  (1.86; 2.34) | 1  2.08  (1.84; 2.35) | 1  2.05  (1.81; 2.31) | 1  1.70  (1.46; 1.97) | 1  2.00  (1.78; 2.24) | 1  1.77  (1.56; 2.01) | 1  1.75  (1.54; 2.00) | 1  1.72  (1.51; 1.95) | 1  1.30  (1.11; 1.52) |
| Hypertension^3^  - No  - Yes | 1  3.12  (2.82; 3.46) | 1  1.90  (1.69; 2.13) | 1  1.88  (1.66; 2.12) | 1  1.85  (1.63; 2.09) | 1  1.66  (1.46; 1.88) | 1  2.47  (2.22; 2.76) | 1  1.73  (1.54; 1.95) | 1  1.68  (1.49; 1.90) | 1  1.66  (1.47; 1.88) | 1  1.49  (1.31; 1.68) |
| High total cholesterol^4^  - No  - Yes | 1  2.51  (2.26; 2.79) | 1  1.76  (1.57; 1.98) | 1  1.74  (1.54; 1.96) | 1  1.75  (1.55; 1.97) | 1  1.65  (1.46; 1.87) | 1  2.12  (1.89; 2.38) | 1  1.59  (1.41; 1.80) | 1  1.55  (1.36; 1.76) | 1  1.54  (1.36; 1.75) | 1  1.44  (1.27; 1.63) |
| High LDL-cholesterol^5^  - No  - Yes | 1  2.10  (1.89; 2.33) | 1  1.56  (1.40; 1.74) | 1  1.54  (1.37; 1.73) | 1  1.54  (1.37; 1.73) | 1  1.44  (1.28; 1.62) | 1  1.76  (1.58; 1.96) | 1  1.42  (1.27; 1.59) | 1  1.39  (1.24; 1.57) | 1  1.39  (1.24; 1.56) | 1  1.29  (1.14; 1.44) |
| Low HDL-cholesterol^6^  - No  - Yes | 1  1.41  (1.27; 1.56) | 1  1.56  (1.41; 1.74) | 1  1.56  (1.40; 1.75) | 1  1.59  (1.43; 1.78) | 1  1.46  (1.30; 1.64) | 1  1.41  (1.27; 1.56) | 1  1.46  (1.31; 1.63) | 1  1.48  (1.32; 1.66) | 1  1.50  (1.33; 1.68) | 1  1.39  (1.24; 1.56) |
| High triglyceride^7^  - No  - Yes | 1  2.16  (1.94; 2.41) | 1  2.01  (1.79; 2.26) | 1  2.06  (1.82; 2.32) | 1  2.09  (1.85; 2.36) | 1  1.90  (1.68; 2.15) | 1  1.96  (1.75; 2.20) | 1  1.88  (1.67; 2.12) | 1  1.87  (1.65; 2.12) | 1  1.88  (1.66; 2.13) | 1  1.71  (1.51; 1.94) |
| Dyslipidemia^8^  - No  - Yes | 1  2.38  (2.08; 2.72) | 1  1.95  (1.70; 2.23) | 1  1.92  (1.66; 2.22) | 1  1.95  (1.69; 2.26) | 1  1.75  (1.51; 2.04) | 1  1.76  (1.54; 2.01) | 1  1.55  (1.35; 1.77) | 1  1.54  (1.34; 1.77) | 1  1.54  (1.34; 1.78) | 1  1.39  (1.21; 1.60) |

^1^BMI categories were based on the WHO cut-offs for Asian Population

^2^Ethnic-Specific (Asian) waist-circumference cut-offs for abdominal obesity were >90 cm for men and >80 cm for women.

^3^Hypertension was defined as systolic blood pressure >140 mmHg AND/OR diastolic blood pressure >90 mmHg OR previous hypertension diagnosis with current use of anti-hypertensive medications.

^4^High total cholesterol (hypercholesterolemia) was defined as total cholesterol levels ≥5.2 mmol/L ((≥200 mg/dL).

^5^High LDL-cholesterol was defined as LDL-cholesterol levels ≥3.4 mmol/L (≥130 mg/dL).

^6^Low HDL-cholesetrol was defined as HDL-cholesterol levels <1.0 mmol/L (<40 mg/dL) in men or <1.3 mmol/L (<50 mg/dL) in women.

^7^Hypertriglyceridemia was defined as triglyceride levels ≥1.7 mmol/L (≥150 mg/dL).

^8^Dyslipidemia was defined based of one or more of the following criteria: total cholesterol ≥200 mg/dL, OR LDL-cholesterol ≥130 mg/dL, OR low triglyceride (≥150 mg/dL), OR low HDL-cholesterol (<40 mg/dL in men or <50 mg/dL in women).

Model 1: adjusted for age and sex.

Model 2: adjusted for model 1 + other socio-demographic determinants (education, employment, marital, and socio-economic status).

Model 3: adjusted for model 2 + lifestyle determinants (physical activity, fruit and vegetable intake, smoking behavior, and alcohol consumption).

Model 4: adjusted for model 3 + body mass index

**Supplementary Table 8. Age, sex, and BMI among the groups generated from the interaction between urban/rural and clinical factors.**

| **Groups** | **Age** | **Sex (%male)** | **BMI** |
| --- | --- | --- | --- |
| **BMI Categories**   - Urban, Underweight - Urban, Normo-weight - Urban, Overweight/Obese - Rural, Underweight - Rural, Normo-weight - Rural, Overweight/Obese | 37.6 (18.6)  41.5 (15.9)  44.1 (13.1)  46.7 (21.4)  44.6 (17.4)  43.7 (14.0) | 64.7 (62.1; 67.3)  60.1 (58.8; 61.4)  42.3 (41.3; 43.3)  62.6 (60.4; 64.8)  62.1 (60.9; 63.2)  35.6 (34.5; 36.7) | 17.1 (1.0)  20.9 (1.2)  27.6 (3.9)  17.2 (1.2)  20.8 (1.3)  27.0 (3.7) |
| **Abdominal Obesity**   - Urban, No abdominal obesity - Urban, Abdominal obesity - Rural, No abdominal obesity - Rural, Abdominal obesity | 40.6 (15.3)  45.5 (13.4)  44.2 (17.3)  45.0 (14.5) | 64.6 (63.6; 65.5)  29.9 (28.7; 31.1)  63.2 (62.4; 64.0)  18.6 (17.4; 19.8) | 21.7 (3.0)  28.3 (4.3)  21.2 (3.1)  27.8 (4.3) |
| **Hypertension**   - Urban, No hypertension - Urban, Hypertension - Rural, No hypertension - Rural, Hypertension | 37.9 (14.0)  49.7 (13.6)  40.2 (16.1)  51.0 (15.1) | 52.8 (51.8; 53.8)  46.6 (45.3; 47.8)  54.9 (54.0; 55.8)  43.8 (42.7; 44.9) | 23.4 (4.3)  25.9 (4.9)  22.2 (4.2)  24.3 (4.9) |
| **Hypercholesterolemia**   - Urban, No hypercholesterolemia - Urban, Hypercholesterolemia - Rural, No hypercholesterolemia - Rural, Hypercholesterolemia | 40.0 (14.9)  48.8 (12.9)  42.4 (16.8)  50.3 (14.3) | 53.4 (52.5; 54.3)  43.0 (41.6; 44.5)  54.0 (53.2; 54.8)  40.8 (39.3; 42.3) | 23.8 (4.7)  25.6 (4.7)  22.7 (4.4)  24.2 (4.9) |
| **High LDL-Cholesterol**   - Urban, Low/normal LDL-cholesterol - Urban, High LDL-cholesterol - Rural, Low/normal LDL-cholesterol - Rural, High LDL-cholesterol | 39.8 (15.1)  47.2 (13.4)  42.3 (17.1)  48.5 (14.8) | 53.0 (52.1; 53.9)  46.0 (44.8; 47.3)  53.9 (53.0; 54.8)  44.3 (43.0; 45.6) | 23.6 (4.6)  25.6 (4.8)  22.5 (4.4)  24.2 (4.9) |
| **Hypertriglyceridemia**   - Urban, No hypertriglyceridemia - Urban, Hypertriglyceridemia - Rural, No hypertriglyceridemia - Rural, Hypertriglyceridemia | 41.3 (15.4)  46.0 (12.9)  43.5 (17.0)  47.2 (15.2) | 46.4 (45.5; 47.3)  60.1 (58.7; 61.5)  48.1 (47.2; 48.9)  57.7 (56.2; 59.2) | 23.8 (4.7)  26.0 (4.4)  22.6 (4.5)  24.4 (4.8) |
| **Low HDL-Cholesterol**   - Urban, High HDL-cholesterol - Urban, Low HDL-cholesterol - Rural, High HDL-cholesterol - Rural, Low HDL-cholesterol | 43.3 (15.3)  41.7 (14.2)  45.8 (16.7)  42.5 (16.3) | 55.2 (54.2; 56.2)  43.1 (41.9; 44.4)  58.4 (57.4; 59.3)  39.3 (38.1; 40.5) | 23.5 (4.5)  25.6 (4.8)  22.3 (4.2)  24.2 (5.0) |
| **Dyslipidemia**   - Urban, No dyslipidemia - Urban, Dyslipidemia - Rural, No dyslipidemia - Rural, Dyslipidemia | 38.8 (15.5)  44.3 (14.3)  42.8 (17.5)  45.2 (16.2) | 55.6 (54.1; 57.0)  48.0 (47.1; 48.9)  60.3 (58.9; 61.8)  45.9 (45.1; 46.8) | 22.5 (4.4)  25.2 (4.7)  21.6 (3.8)  23.8 (4.8) |

Data were presented as mean (standard deviation) for age and BMI variables; meanwhile proportion and its 95% confidence interval for sex variable.

BMI: body mass index
